# A Novel Reliable and Efficient Procedure for Purification of Mature Osteoclasts Allowing Functional Assays in Mouse Cells

**DOI:** 10.3389/fimmu.2018.02567

**Published:** 2018-11-02

**Authors:** Maria-Bernadette Madel, Lidia Ibáñez, Matthieu Rouleau, Abdelilah Wakkach, Claudine Blin-Wakkach

**Affiliations:** ^1^CNRS UMR7370, Laboratoire de PhysioMédecine Moléculaire, Faculty of Medicine, Nice, France; ^2^University Nice Sophia Antipolis, Nice, France

**Keywords:** Osteoclasts isolation, multinucleated cells, nuclei staining, monocytes, flow cytometry, cell sorting, osteoimmunology

## Abstract

Osteoclasts (OCLs) are multinucleated phagocytes of monocytic origin responsible for physiological and pathological bone resorption including aging processes, chronic inflammation and cancer. Besides bone resorption, they are also involved in the modulation of immune responses and the regulation of hematopoietic niches. Accordingly, OCLs are the subject of an increasing number of studies. Due to their rarity and the difficulty to isolate them directly *ex vivo*, analyses on OCLs are usually performed on *in vitro* differentiated cells. In this state, however, OCLs represent a minority of differentiated cells. Since up to date a reliable purification procedure is still lacking for mature OCLs, all cells present in the culture are analyzed collectively to answer OCL-specific questions. With the development of in-depth transcriptomic and proteomic analyses, such global analyses on unsorted cells can induce severe bias effects in further results. In addition, for instance, analysis on OCL immune function requires working on purified OCLs to avoid contamination effects of monocytic precursors that may persist during the culture. This clearly highlights the need for a reliable OCL purification procedure. Here, we describe a novel and reliable method to sort OCLs based on cell multinucleation while preserving cell viability. Using this method, we successfully purified multinucleated murine cells. We showed that they expressed high levels of OCL markers and retained a high capacity of bone resorption, demonstrating that these are mature OCLs. The same approach was equally applied for the purification of human mature OCLs. Comparison of purified OCLs with mononucleated cells or unsorted cells revealed significant differences in the expression of OCL-specific markers at RNA and/or protein level. This exemplifies that substantially better outcomes for OCLs are achieved after the exclusion of mononucleated cells. Our results clearly demonstrate that the in here presented procedure for the analysis and sorting of pure OCLs represents a novel, robust and reliable method for the detailed examination of bona fide mature OCLs in a range that was previously impossible. Noteworthy, this procedure will open new perspectives into the biology of osteoclasts and osteoclast-related diseases.

## Introduction

Osteoclasts (OCLs) are multinucleated cells of monocytic origin responsible for bone resorption (1). They represent a heterogeneous population of cells having all the capacity to resorb bone but that differ in their effects on immune responses, depending on their environment (2–4). Alterations of their function are involved in a number of bone-related pathologies such as osteoporosis, rheumatic diseases, inflammatory bowel disease and other chronic inflammatory diseases or cancer, making OCLs an important matter of investigations. These are mainly focused on OCL differentiation, on OCL resorptive and immune functions, on the identification of OCL-specific markers and on strategies for inhibiting bone resorption. Such analyses increasingly require novel potent and highly sensitive technologies for genomic, transcriptomic and proteomic in-depth analyses that necessitate working on purified populations. As a result, nowadays the capacity to purify OCLs represents a major goal that has not yet been satisfactorily achieved.

OCLs are defined as multinucleated monocytic cells, expressing the tartrate resistant-acid phosphatase (TRAcP) and resorbing the bone matrix. As other cells of monocytic origin (5), OCLs display a phenotypic and functional heterogeneity depending on their precursors and environment (2). Except in chicken and rabbit, OCLs cannot be isolated directly *ex vivo* in sufficient quantities to perform further analyses due to their rarity (6, 7). Therefore, much of their biology has been established by studies using *in vitro*-generated OCLs derived from different precursors, in particular in mice and humans (7–9). Fully differentiated OCLs account for only a small proportion of total cells present in the culture (<20%) (2) that varies according to the differentiation conditions.

Such a heterogeneity is well-known after *in vitro* differentiation of monocytic cells such as monocytes and dendritic cells and it is no longer conceivable to further analyse these cells without prior purification (5). In contrast, most of the studies using *in vitro*-differentiated OCLs are performed without any purification step. Indeed, to date, a reliable methodology for OCL purification is still missing. This is mainly due to their large size, which makes it difficult to handle mature OCLs, as well as due to the lack of specific surface markers to distinguish OCLs from other cells present in the culture. Thus, since the abundant remaining mononucleated cells have been exposed to osteoclastogenic conditions, they are quite simply considered as OCLs that have not (yet) fused and are generally analyzed together with mature OCLs as one pool of cells. However, up to now, it has not clearly been evinced whether these mononucleated cells are indeed equivalent to OCLs in terms of gene and protein expression. This point is particularly important because if they differ from mature OCLs, these contaminant cells are likely to induce bias in further analysis. Yet, some studies used enriched OCLs for example by selective enzymatic detachment from the culture plates based on their stronger adherence capacity compared to mononucleated cells (10). Nonetheless, such approaches do not allow a high degree of purification, which can considerably differ depending on the procedure used and may be difficult to evaluate. As the striking feature of OCLs is their multinucleation, we previously established a procedure based on DAPI staining of the nuclei to isolate multinucleated OCLs using flow cytometry. However, this procedure necessitates cell fixation that prevents further functional analysis (2). This could be overcome using an indirect FACS gating strategy on unfixed cells, but this reduced the yield and reliability of OCL purification (2). Therefore, our aim was to set up a reliable, quantifiable and efficient procedure to purify living mature OCLs and to validate the benefits of this procedure by analysing some OCL-specific characteristics.

Using Hoechst 33342 staining, OCLs were isolated on a cell sorter based on their multinucleation. We showed that this procedure preserved the viability, phenotypic as well as functional properties of murine mature OCLs. Additionally, we demonstrated that the expression of OCL-specific markers was greatly increased when comparing sorted OCLs to unsorted cells. Furthermore, we also showed that the mononucleated cells present in the culture express very low levels of OCL markers and that the presence of these cells significantly altered RNA and protein expression profiles during the analysis of OCL-specific markers. The same procedure was applied for the isolation of human OCLs and despite it didn't allow functional assay because of low cell viability, it greatly improved analysis of OCL-specific markers using flow cytometry. Based on these data, this innovative and straightforward method represents a reliable procedure for the purification of bona fide mature OCLs. Our results clearly emphasize the importance of excluding the interfering mononucleated cells from OCLs to enable new insights into the biology of osteoclasts and the field of osteoimmunology.

## Materials and methods

### Reagents and chemicals

Minimum Essential Medium (MEM) Alpha Medium with Ribonucleosides and Deoxyribonucleosides, Penicillin/Streptomycin (10,000 U/ml Penicillin 10,000 μg/ml Streptomycin), 2-Mercaptoethanol 50 mM, Fetal Bovine Serum (FBS), ethylenediaminetetraacetic acid (EDTA), Nunc^TM^ Lab-Tek^TM^ II Chamber Slides, glass cover slips (Menzel Deckgläser, 24 × 50 mm), anti-human CD44-PercP-Cyanine 5.5 (clone IM7), Annexin V-Biotin Apoptosis Detection Kit, anti-mouse APC-Streptavidin, Alexa Fluor® 488 goat anti-rat IgG (H+L; 2 mg/ml) as well as Nanodrop 2,000 spectrophotometer, TRIzol Reagent, Superscript II Reverse Transcriptase (200 U/μl), Random Hexamers (50 μM, 5 nmoles), RNaseOUT Ribonuclease Inhibitor (40 U/μl), 0.1 M DTT, 5x First Strand Buffer and the StepOnePlusTM Real Time PCR System Thermal Cycling Block were purchased from ThermoFisher Scientific (Illkirch, France). Ficoll Paque^TM^ Plus and characterized HyClone^TM^ Fetal Bovine Serum came from GE Healthcare (Buc, France). C57Bl/6J OlaHsd mice were purchased from Envigo (Gannat, France). Mouse RANK-L and M-CSF were from R&D Systems (Lille, France) while human RANK-L and M-CSF were from PeproTech (Neuilly-sur-Seine, France). Accutase Solution, bisBenzimide H 33342 trihydrochloride (Hoechst 33342) ≥98% HPLC and TLC, Tween® 20, TRAP Kit, Alizarin Red S, 4′,6-diamidino- 2-phénylindole (DAPI), Acetone, Dulbecco's Phosphate Buffered Saline Modified, without calcium chloride and magnesium chloride (DPBS) as well as Red Blood Cell Lysing Buffer Hybri-MaxTM and ImmunoHistoMount were purchased from Sigma-Aldrich (Saint-Quentin Fallavier, France). Anti-Biotin MicroBeads were bought from Miltenyi Biotec (Paris, France). Osteo-Assay plates, 96 well with flat bottom were from Corning (New York, United States) and RNase-free water was obtained from Macherey-Nagel (Düren, Germany). The Axio Observer D1 confocal microscope and the PrimoVert phase-contrast microscope came from Carl Zeiss (Marly le Roi, France). Biotinylated anti-mouse CD51 (clone RMV-7), and CD11b (clone M1/70) monoclonal antibodies, mouse anti-human CD51/61-FITC (clone 23C6), rat anti-mouse CD51-PE (clone RMV-7), FITC-streptavidin conjugate as well as the FACS Canto II and the FACS Aria IIu were purchased from BD Bioscience (Le Pont de Claix, France). The SensiFAST SYBR HI-ROX Kit and primers for RT-qPCR (mouse *Calcr, Acp5, ItgB3, Mmp9, Ctsk, Atp6V03a/Tcirg1, 36B4* and human *ACP5, MMP9, CLCN7, CTSK*, and *36B4*) were bought from BIOLINE (Paris, France) and Eurofins Genomics (Les Ulis, France), respectively.

### Osteoclast generation

Eight-week old C57BL/6JOlaHsd male mice were purchased from Envigo and maintained under a 12-h light/12-h dark cycle with free access to water and standard mouse diet. Animals were housed at 4 mice per cage in our animal facility in accordance with the general guidelines of the institute. Approval for their use in this study was obtained from the Institutional Ethic Committee for Laboratory Animals (CIEPAL-Azur). Murine bone marrow cells were obtained by flushing the long bones with PBS 1X and subsequent lysis of the red blood cells (Sigma-Aldrich) as described (11). Murine CD11b^+^ monocytes were isolated from these bone marrow cells using microbeads for magnetic separation according to the manufacturer's protocol (Miltenyi Biotec) and cultured as described previously (2). Briefly, 2 × 10^5^ CD11b^+^ monocytes/well were seeded in 24-well plates in 500 μL OCL differentiation medium (α-MEM supplemented with 5% FBS (Hyclone, GE Healthcare), 1% penicillin-streptomycin, 50 μM 2-mercaptoethanol) plus murine RANK-L (30 ng/ml) and M-CSF (25 ng/ml) (R&D Systems) for 5 days. Human whole blood from healthy platelet donors was purchased from the Etablissement Français du Sang (EFS) to which donors gave informed consent. Human peripheral blood mononuclear cells (PBMCs) were isolated by density centrifugation over Ficoll Paque Plus as described (12, 13). A total of 5 x 10^5^ PBMCs/well were seeded in 24-well plates in 500 μL OCL differentiation medium containing human RANKL (50 ng/ml) and M-CSF (30 ng/ml) (PeproTech) for 7 days. Medium was changed every 3–4 days.

### Osteoclast sorting and FACS analysis

Differentiated OCLs were detached using Accutase (Sigma-Aldrich). Therefore, medium was removed from the 24-well plates and each well was washed with 500 μl PBS 1X before incubation with 300 μl Accutase at 37°C for about 20–30 min. Subsequently, cells were carefully collected and transferred into a new falcon tube containing Fetal Bovine Serum (FBS, ThermoFisher Scientific). Plates were washed extensively with PBS 1X and added to collected cells. Detached cells were spun down at 350 g for 5 min and labeled with 5 μg/ml Hoechst 33342 (Sigma-Aldrich) in PBS 1X supplemented with 1% FBS and 2 mM EDTA (PSE) for 30 min at 37°C to analyze the cells based on their nuclei number. Cells were spun down at 350 g for 5 min and re-suspended at 6 × 10^6^ cells/ml in ice-cold PSE for cell sorting. At this point, samples may be directly sorted or further stained with antibodies. All subsequent manipulations that follow the Hoechst staining process were strictly performed on ice in order to prevent further dye efflux. Before FACS analysis, cells were filtered on a 100 μm nylon mesh. This procedure requires a violet laser (ex-405 nm, band pass filter 440/450 nm) on the FACS or cell sorter to detect Hoechst 33342 (ex/em: 361/497 nm).

After doublet exclusion (see results section), singlets with 1–2 nuclei and ≥3 nuclei were selected by a histogram display and by plotting against the nuclei number. Cells were sorted on a FACS Aria IIu (BD Bioscience) using a 100 μm nozzle at a flow rate of 2000 events/s and a sorting efficacy between 95 and 100%. Sorted osteoclasts were collected in FBS.

For FACS analysis, Hoechst-stained cells were subsequently labeled with human anti-CD51/61 (10 μl/test; 23C6), human anti-CD44 (3 μl/test; IM7), murine anti-CD51 (1:200; RMV-7) and murine anti-calcitonin receptor (CTR; 1:100; kind gift from Prof. P Sexton, Monash University) antibodies in PSE for 15 min on ice protected from light. Following anti-CTR incubation, samples were labeled with Alexa488-anti rat IgG (1:100) in PSE for 15 min on ice covered from light. After washing with 2 ml ice-cold PSE, cells were analyzed in a total volume of 300 μl PSE on a FACS Canto II using the same gating strategy and doublet discrimination as specified for cell sorting. Flow cytometry data were analyzed using FlowJo X 10.0.7 (FlowJo, LLC).

### Cell viability assay

Cell viability was analyzed by the Annexin-V/Propidium Iodine (PI) apoptosis assay. Briefly, human and mouse OCLs were differentiated and collected as described above. Cells were incubated in the presence or absence of 5 μg/ml Hoechst 33342 for 30 min at 37°C. After washing, cells were either sorted based on their nuclei expression prior to viability test or directly stained with Annexin-V/PI using the Annexin V-Biotin Apoptosis Detection Kit (ThermoFisher Scientific) and APC-labeled Streptavidin (1:500) following manufacturer's instructions. Flow cytometry was used to quantify viable cells (Annexin-V^neg^/PI^neg^ cells).

### Immunofluorescence

OCLs were differentiated as described above on 8-well Nunc^TM^ Lab-Tek^TM^ II chamber slides in 300 μl osteoclast differentiation medium. After 5 days, cells were fixed with 100 μl acetone for 1 min at room temperature, rinsed with washing buffer (PBS 1X containing 0.05% Tween 20 and 1% FBS), and incubated with biotinylated anti-CD51 (1:150) or anti-calcitonin receptor (1:200; kind gift from Prof. P Sexton, Monash University, Australia) in incubation buffer (PBS 1X containing 0.05% Tween 20, 1% FBS and 5% goat serum) for 1 h at room temperature covered from light. Subsequently, cells were rinsed with washing buffer followed by FITC-streptavidin (1:75) or FITC-anti rat IgG (1:100) staining for 45 min at room temperature in the dark. After washing with washing buffer, nuclei were counterstained using 1 μg/ml DAPI (Sigma-Aldrich) in PBS 1X for 5 min at room temperature protected from light and washed twice with washing buffer. Slices were mounted with ImmunoHistoMount (Sigma-Aldrich). Fluorescence microscopy analysis of cells was performed with an Axio Observer D1 microscope (Zeiss) and pictures were taken using AxioVision Rel. 4.8 software (Zeiss). Images were processed using Fiji/ImageJ software (14).

### TRAcP staining and functional analysis

A total of 2 × 10^4^ sorted OCLs, sorted 1-2N cells or unsorted differentiated cells was seeded per well on 48-well plates in 300 μL α-MEM containing 10% FBS (Hyclone, Perbio) plus 30 ng/ml mouse RANK-L. After overnight incubation, medium was removed and cells were fixed in 100 μl fixation solution (containing 60% acetone and 40% citrate solution, as recommended by the manufacturer) for 30 s. Tartrate-resistant acid phosphatase (TRAcP) activity was analyzed using the leucocyte acid phosphatase kit in the presence of tartrate according to manufacturer's instructions (Sigma-Aldrich). Using phase-contrast microscopy, TRAcP^+^ cells with ≥3 nuclei were enumerated as OCLs. For time course analysis, cells were kept in culture for up to 5 days after sorting, before TRAcP staining.

Matrix dissolution activity was evaluated by seeding a total of 10^4^ unsorted cells or sorted 1-2N cells and OCLs on 96-well osteoassay plates (Corning) in 200 μl α-MEM containing 10% FBS (Hyclone, Perbio) and 30 ng/ml murine RANK-L. After overnight incubation, medium was aspirated and cells were removed by the addition of 200 μl distilled sterile water. Calcified matrix was stained for 1 min with 2% Alizarin Red and washed with PBS 1X. Resorbed areas were quantified using Fiji/ImageJ software (14). In addition, bone resorption capacity was tested by seeding unsorted cells or sorted 1-2N cells and OCLs on dentin slices in 96-well plates as stated above. To detect resorption lacunae, cells were removed from the dentin by sonification and dentin slices were stained with 1% toluidine blue as described previously (9). Resorption lacunae were analyzed using light microscopy.

### RT-qPCR

Total RNA of human and mouse cells was extracted just after cell sorting using TRIzol reagent with subsequent isopropanol precipitation according to manufacturer's specifications. Isolated RNA was quantified using a Nanodrop 2000 spectrophotometer (ThermoFisher Scientific). After reverse transcription (Superscript II, Life Technologies) RT-qPCR was performed as described (15) using the following primers: *Calcr (calcitonin receptor)* (5′-CTTCCATGCTGATCTTCTGG-3′; 5′-CAGATCTCCATTGGGCACAA-3′), *Acp5* (TRAcP) (5′-TGCCTACCTGTGTGGACATGA-3′; 5′-CACATAGCCCACACCGTTCTC-3′), *ItgB3 (vitronectin receptor chain)* (5′-CTTTGACGCCATCATGCAG-3′; 5′-TATGGGTCTTGGCATCCGT-3′), *Mmp9* (5′-TGAGTCCGGCAGACAATCCT-3′; 5′-CGCCCTGGATCTCAGCAATA-3′), *Ctsk* (5′-CAGCAGAGGTGTGTACTATG-3′; 5′-GCGTTGTTCTTATTCCGAGC-3′), *Atp6V0a3/Tcirg1* (5′-CGCTGCGAGGAACTGGAG-3′; 5′-AGCGTCAGACCTGCCCG-3′) for murine samples and *ACP5* (TRAcP) (5′-GACCACCTTGGCAATGTCTCTG-3′; 5′-TGGCTGAGGAAGTCATCTGAGTTG-3′), *MMP9* (5′-GAGACGCCCATTTCGACGA-3′; 5′-TCGAAGATGAAGGGGAAGTG-3′), *CTSK* (5′-TGAGGCTTCTCTTGGTGTCCATAC-3′; 5′-AAAGGGTGTCATTACTGCGGG-3′), *CLCN7* (5′-GATCGTGGGCGACGTCTT-3′; 5′-AGTGCAGGAAGGGCACACTCT-3′) for human samples. Real-time quantitative PCR was performed on a StepOne Plus real-time PCR instrument (ThermoFisher Scientific) using an initial denaturation and polymerase activation step at 95°C for 2 min followed by 40 cycles of denaturation for 3 s at 95°C and primer annealing/extension for 30 s a 60°C. Samples of three independent experiments were run in triplicates and results were normalized to the *36B4* RNA. Data analysis was carried out using StepOne Software v2.3 (ThermoFisher Scientific) and assessed using the 2^−ΔCt^ method as described (16).

### Statistical analysis

Statistical analysis was performed using GraphPad Prism 7.0. Data are presented as mean ±SEM of at least three biological replicates. Error bars for human and mouse gene expression analysis by RT-qPCR indicate the mean with 95% confidence interval. Statistical significance was determined using student's *t*-test with Bonferroni adjustment for multiple testing with a *p* < 0.05.

## Results and discussion

### FACS sorting and analysis strategy for mouse and human osteoclasts

**Figure 1:**
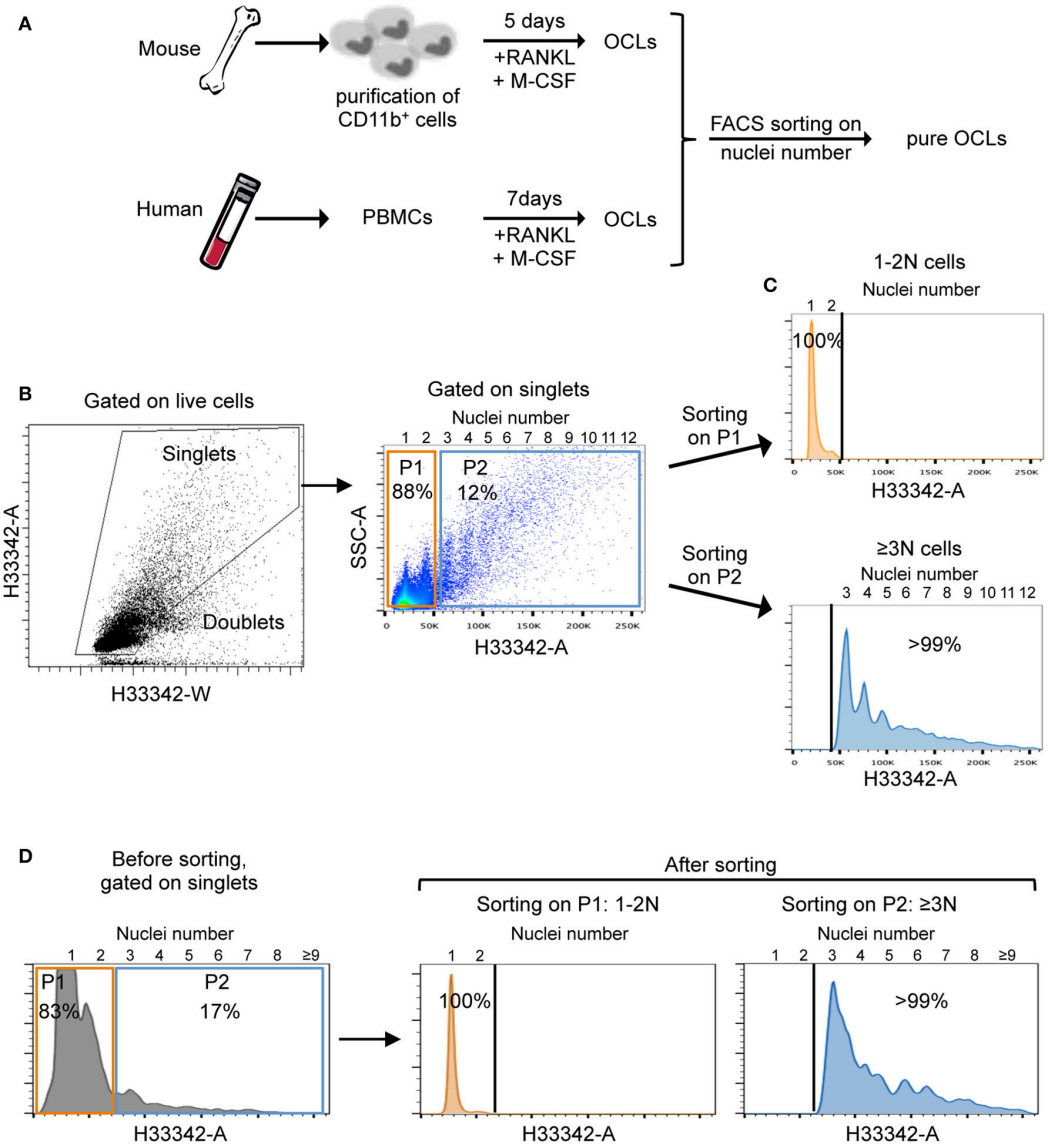
Osteoclast preparation and cell sorting strategy. **(A)** Schematic representation of OCL preparation from mouse bone marrow progenitors and human peripheral blood mononuclear cells (PBMCs). **(B)** Gating strategy for FACS analysis and sorting of ≥3N cells using Hoechst 33342 after doublet discrimination. Nuclei numbers are indicated at the top of FACS graphs. **(C,D)** Representative cell sorting of 1–2N cells (orange) and ≥3N cells (blue) using flow cytometry for murine **(C)** and human **(D)** cells. After gating on single cells, 1–2N cells (P1, orange) and ≥3N (P2, blue) cells were selected for sorting. To validate the gating strategy and the sorting process, sorted cells were reanalyzed in a flow cytometer. Sorted cells with 1–2N and ≥3N revealed >99% positive events with corresponding nuclei number.

In order to reliably and effectively purify them, mature OCLs were differentiated from murine bone marrow CD11b^+^ cells and human PBMCs (Figure 1A). After the differentiation, cells were detached and their nuclei were stained with the vital dye Hoechst 33342 before FACS sorting. The flow rate and nozzle aperture of the cell sorter were chosen to adapt to the big cell size and maintain an appropriate laminar flux, as described in the Material and Methods section. After selection of live cells on the forward (FSC) and side scatter (SSC) density plots, multinucleated cells were discriminated from doublets and clumps in accordance with common FACS gating strategies used for cell cycle analysis (17–19). Cells passing through the FACS laser beam are recorded for the time duration (width) and the maximum intensity (height) of the pulse, and for the area under the curve generated by plotting the width against the height. Cell doublets that take longer to pass through the laser beam are recognized with double the width but the same height as a single cell. In contrast, dividing cells having double DNA content show the same width but double the height of a single cell in G0/G1 phase. Osteoclasts have both a higher height (due to multinucleation) and a higher width because of their huge cell size. Thus, using the area and width FACS parameters on the linearly scaled, Hoechst 33342 channel allows to distinguish between doublets/clumps, cells with 1 or 2 nuclei (1-2N cells) and multinucleated cells with 3 and more nuclei (≥3N cells) pursuant to the definition of an OCL (Figure 1B, left panel).

Gating on the mouse ≥3N cell fraction confirmed the initial assumption that only a small proportion of the total cells (approximately 10%) was presented with ≥3 nuclei while about 90% of the cells had one or two nuclei. Interestingly, this strategy revealed that the number of nuclei linearly increased with the size and granularity of the cells (Figure 1B, right panel) as usually observed by microscopy analysis. Further flow cytometry analysis on the sorted 1-2N and ≥3N cell fractions demonstrated the accuracy of this novel method and the high purity of multinucleated cells in the sorted ≥3N cell fraction (Figure 1C), with a majority of sorted cells having between 3 and 8 nuclei. Very large OCLs (more than 10–15 nuclei) were difficult to analyze because they were hardly detached from the plates. Lastly, we applied the same strategy to human cells to isolate multinucleated (≥3N) cells with purity levels equivalent to the ones obtained with murine cells (Figure 1D).

Of note, the mean number of OCLs obtained after sorting corresponded to about 1% of total cells seeded at the beginning of the differentiation (mean of 10 independent experiments). Therefore, contrasting with previous enrichment approaches, this procedure represents a quantifiable and highly reliable approach to isolate multinucleated cells.

### Sorted murine multinucleated cells are living osteoclasts

**Figure 2:**
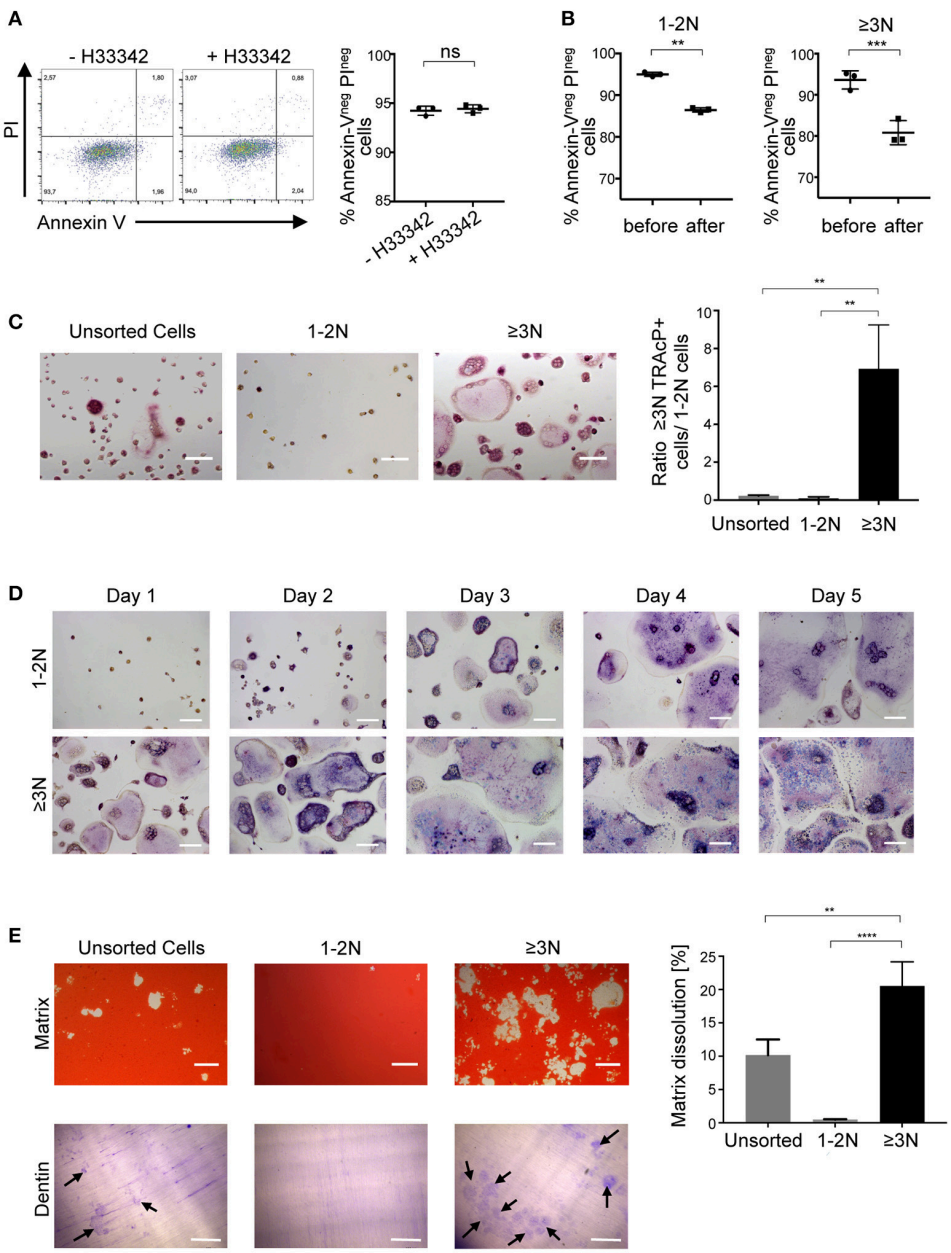
Sorted cells are pure, functioning and living OCLs. **(A)** Cell viability of differentiated murine cells incubated in the presence or absence of 5 μg/ml H33342 was determined by an Annexin-V/PI staining. Annexin-V^neg^ PI^neg^ cells were considered as viable cells and quantified using flow cytometry. **(B)** H33342^+^ cells were gated on 1-2N and ≥3N cells before and after cell sorting to define cell viability using flow cytometry. **(C,D)** TRAcP staining of unsorted and sorted 1-2N and ≥3N cells seeded at the same cell density, 24 h after sorting **(C)** as well as in a 5-day follow-up for 1-2N and ≥3N cells **(D)**. **(E)** Matrix dissolution activity and bone resorption capacity for unsorted cells and sorted 1–2N and ≥3N cells seeded at the same density. Scale bar = 100 μm. Bar graphs show the means ±SEM of three wells in at least three independent experiments. Black arrows = resorption lacunae. ns, not significant; **p < 0.01; ***p < 0.001; ****p < 0.0001.

We then assessed the viability and survival of murine sorted cells. Apoptosis assay revealed that cell viability is not affected by Hoechst 33342 staining (Figure 2A). Moreover, cell viability was equivalent between 1-2N and >3N cells before sorting and this viability was only slightly decreased after cell sorting in both populations (Figure 2B). To further evaluate whether the selected ≥3N cells indeed corresponded to a pure population of OCLs, murine unsorted cells, sorted 1-2 N cells and sorted ≥3N cells were plated at the same cell density and stained for their TRAcP activity. This analysis confirmed that, compared to unsorted cells, in which the proportion of multinucleated cells is low (about 10%, Figure 1B), sorted ≥3N cells corresponded to multinucleated cells of different sizes that are all expressing TRAcP (Figure 2C). Admittedly, very few mononucleated cells can remain in the sorted ≥3N cell fraction likely due to the formation of few doublets between mononucleated cells and OCLs during the FACS acquisition. However, due to the difference in cell size, such doublets cannot be discriminated by the cytometer and excluded by the gating strategy nor by any other previously reported strategy. Therefore, optimization of the sample preparation, addition of EDTA in the buffer used for FACS analysis and filtering of the cells before FACS analysis are crucial to minimize such doublets. As expected, sorted 1-2N cells did not contain multinucleated cells (Figure 2C).

Cell survival and the fate of sorted cells was assessed through a time course analysis. Murine sorted 1-2N cells and ≥3N cells survived at least 5 days after seeding confirming that cell sorting did not compromise cell viability (Figure 2D). Within these 5 days, sorted ≥3N cells continued to fuse to form larger osteoclasts. Part of sorted 1-2N cells were also able to fuse and form multinucleated cells, revealing that part of them had osteoclastogenic capacity (Figure 2D).

To investigate their resorption capacity, sorted ≥3N cells, sorted 1-2N cells and unsorted cells were plated at the same density on a mineralized matrix (Figure 2E, upper panels). Resorbed areas were detected after overnight incubation again confirming that the sorting did not alter OCL viability and resorptive capability. As expected, the resorbed area was considerably higher for sorted ≥3N cells than for unsorted cells confirming the huge difference in the number of plated mature OCLs in the two conditions. No resorption was observed after overnight incubation of 1-2N cells, confirming that they do not correspond to mature OCLs. The same approach was used to evaluate the bone resorption capacity of sorted and unsorted cells after seeding on dentin slices (Figure 2E, lower panels). Resorption pits were not observed for 1-2N cells and were more abundant for ≥3N cells than for unsorted cells, confirming that they are mature OCLs.

These results demonstrate that compared to our previously described protocol for murine OCL sorting using flow cytometry, which was based on an indirect gating strategy according to the cell size and granularity (2), this new procedure enables to analyse murine living and pure bona fide mature OCLs.

### Using the FACS sorting and analysis strategy for OCLs improves the analysis of OCL-specific characteristics

**Figure 3:**
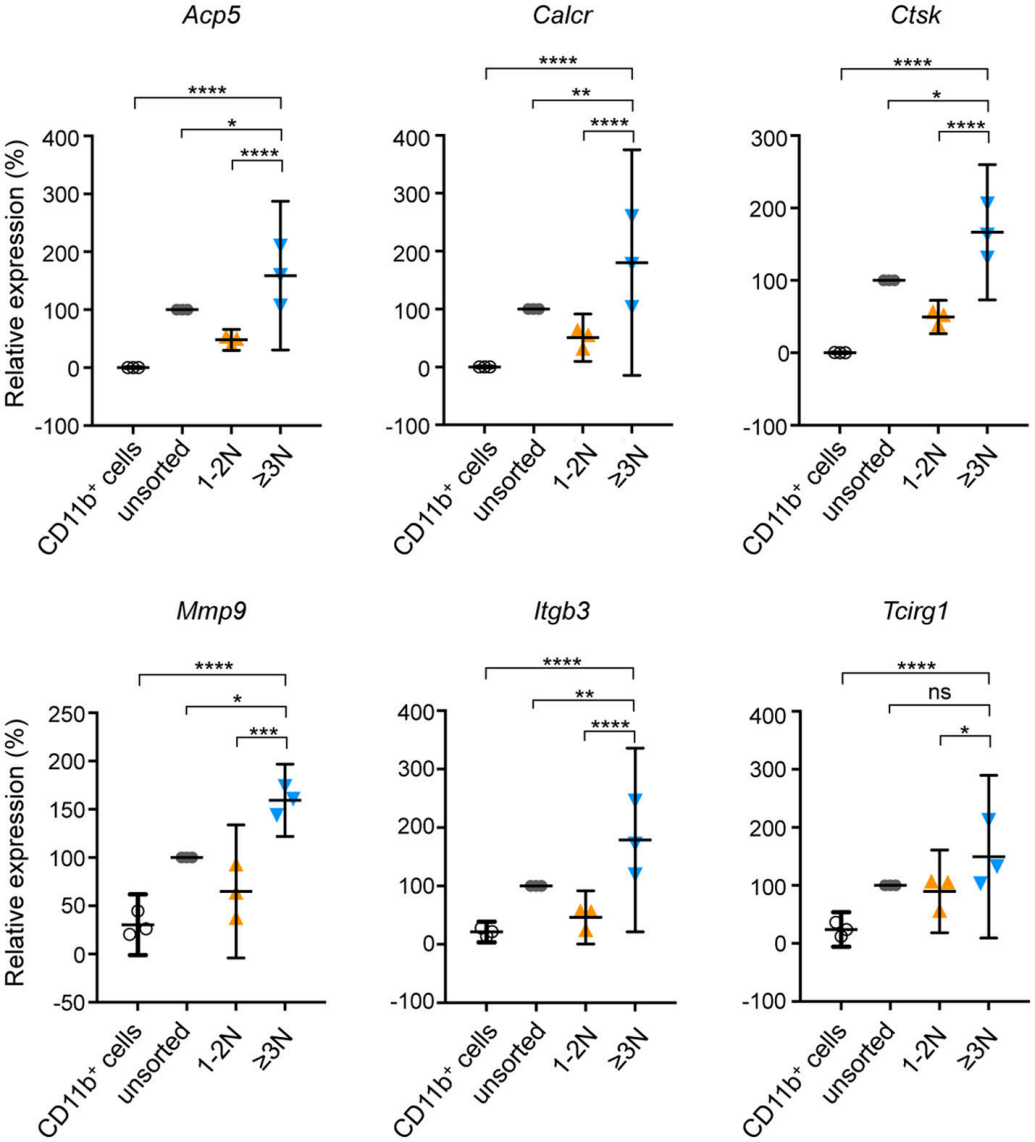
mRNA expression of OCL-specific genes on sorted and unsorted populations. Relative gene expression of *Acp5, Calcr, Ctsk, Mmp9, Itgb3* and *Tcirg1* was determined by RT-qPCR on murine CD11b^+^ OCL precursors (black), unsorted cells (gray), sorted cells with 1-2 nuclei (1–2N, orange) and sorted cells with 3 and more nuclei (≥3N, blue). Unsorted cells were defined as 100% of relative expression and compared to sorted cells. Differences were calculated with the 2^−ΔCt^ method after normalization to the 36B4 RNA. Results are represented as the mean with 95% confidence interval of three independent cell preparations and sortings, each from 1 mouse, and experiments were conducted in triplicates. *Acp5*, tartrate-resistant acid phosphatase; *Calcr*, calcitonin receptor; *Ctsk*, cathepsin K; *Mmp9*, matrix metallopeptidase 9; *Itgb3*, integrin beta 3 of the vitronectin receptor; *Tcirg1*, V-ATPase subunit a3. ns, not significant; *p < 0.05; **p < 0.01; ***p < 0.001; ****p < 0.0001.

We further determined the benefit provided by this procedure on the analysis of OCL specific properties by comparing their expression levels of well-established OCL markers. Real-time quantitative PCR analysis revealed that, compared to their bone marrow CD11b^+^ precursor cells, sorted murine ≥3N cells expressed very high RNA levels of *Tcirg1* (a3 subunit of the V-ATPAse), *Calcr* (calcitonin receptor), *Acp5* (TRAcP), *Mmp9* (matrix metalloproteinase 9), *Itgb3* (Integrin-β3) and *Ctsk* (cathepsin K) (Figure 3) demonstrating that sorted multinucleated cells have the molecular signature of mature OCLs (3, 20, 21). Importantly, sorted 1-2N cells expressed substantially and significantly lower levels of all OCL markers than sorted mature ≥3N OCLs. In the unsorted cell fraction, the expression level of all OCL markers did not significantly differ from the one of the 1-2N cell fraction (Figure 3). As the 1-2N cells represent the vast majority of cells before sorting, their gene expression profile massively overlays the one obtained for the under-represented OCLs. This result demonstrates that the 1-2N cells that persist at the end of the OCL differentiation differ from mature OCLs. It also strongly supports the immense interest of working on purified OCLs when analyzing transcriptomic profiles.

**Figure 4:**
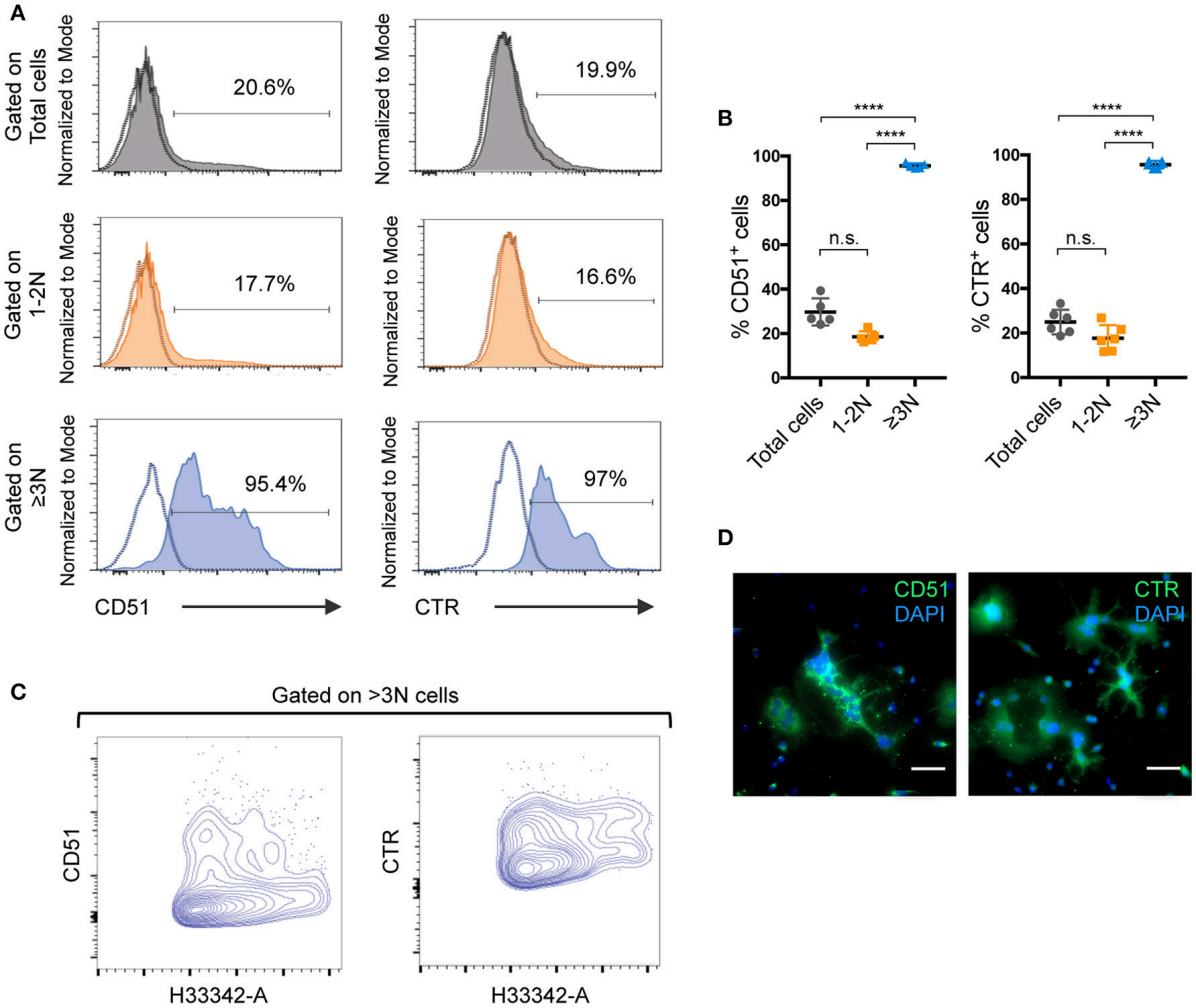
Analysis of pure ≥3N cells significantly improves OCL-specific outcomes for mouse differentiated cells. **(A)** Flow cytometry analysis and **(B)** quantification of CD51 and calcitonin receptor (CTR) positive cells. Using the same gating strategy as for cell sorting, total (gray) and 1–2N (orange) cells were compared to ≥3N cells (blue). Individual negative controls of each population are indicated as dotted lines in respective single parameter histograms and percentage of positive events is indicated. Data are presented as mean ±SEM of at least 5 independent cell preparations, each from 1 mouse. **(C)** FACS contour plot analysis on ≥3N cells for CD51 and CTR by plotting the nuclei number (H33342-A) against CD51 and CTR respectively. **(D)** Immunofluorescence analysis for CD51 and CTR on differentiated mouse cells. Nuclei were stained with DAPI. Scale bar = 50 μm. ns, not significant; ****p < 0.0001.

To better characterize how the presence of 1-2N cells affects the analysis of OCL-specific characteristics, the nuclear staining strategy developed for sorting was applied for a comparative FACS analysis of total murine unsorted cells, 1-2N cells and ≥3N cells. Using the same gating strategy as for OCL sorting, we analyzed the expression of two OCL markers, CD51 (vitronectin receptor α chain, integrin αV) and calcitonin receptor (CTR) (20–22). Specific gating on ≥3N cells showed that almost 100% of these cells expressed CD51 and CTR (Figures 4A,B), as expected for bona fide OCLs. In contrast, <20% of the cells were positive for these markers when gating on the 1-2N cell fraction or on total cells. These results revealed that excluding the predominant 1-2N cells from the analysis enabled the detection of positive events from the under-represented OCLs as well as an appropriate assessment of their protein expression levels. These findings confirmed that the 1-2N cells significantly differ from ≥3N OCLs not only in their RNA but also in their protein expression pattern. Moreover, FACS gating on ≥3N cells revealed that OCLs harbor both high and intermediate expression levels of CTR and CD51 (Figure 4A lower panels), which are independent of the nuclei number (Figure 4C). This was likewise observed by immunofluorescence microscopy on fully differentiated OCLs (Figure 4D). However, this interesting information was completely lost when analyzing total cells because the signals from the 1-2N cells dramatically overlay those from mature OCLs. Thus, focused analysis of ≥3N cells may reveal new specific OCL characteristics that were forsaken up to now due to non-specific signals arising from the contaminating 1-2N cells.

**Figure 5:**
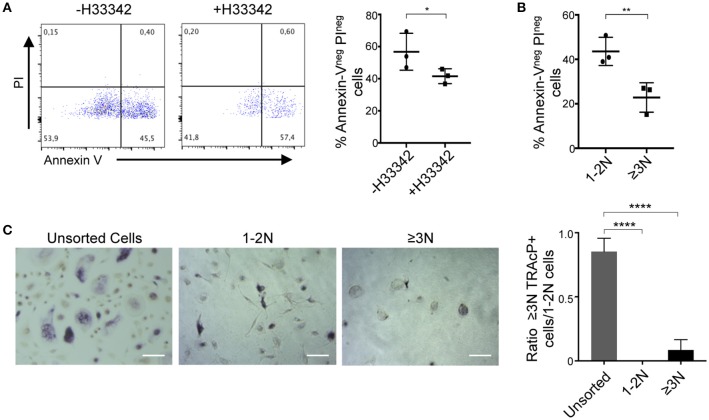
Analysis of sorted human osteoclasts**. (A,B)** Viability assay on differentiated human cells by Annexin-V/PI staining. **(A)** Collected cells were incubated with or without 5 μg/ml H33342 and Annexin-V^neg^ PI^neg^ cells were quantified by flow cytometry. **(B)** Cell viability assay after gating in 1–2N and ≥3N cells. **(C)** TRAcP staining of unsorted and sorted 1–2N and ≥3N cells 24 h after FACS sorting and seeded at the same cell density. *p < 0.05; **p < 0.01; ****p < 0.0001.

Next, we investigated the sorting and analysis approach for human cells. The same sorting and gating strategy as applied for murine cells was used to compare human ≥3N OCLs with the 1-2N cells growing together with them. Contrasting with what was observed for murine OCLs, Hoechst 33342 staining strongly reduced human cell viability (Figure 5A). Human ≥3N cells were more affected by this procedure than 1-2N cells (Figure 5B) and did not survive after overnight incubation (Figure 5C) demonstrating very different behavior compared to murine OCLs. Therefore, further optimization of this procedure is necessary to allow functional analysis on sorted human OCLs.

**Figure 6:**
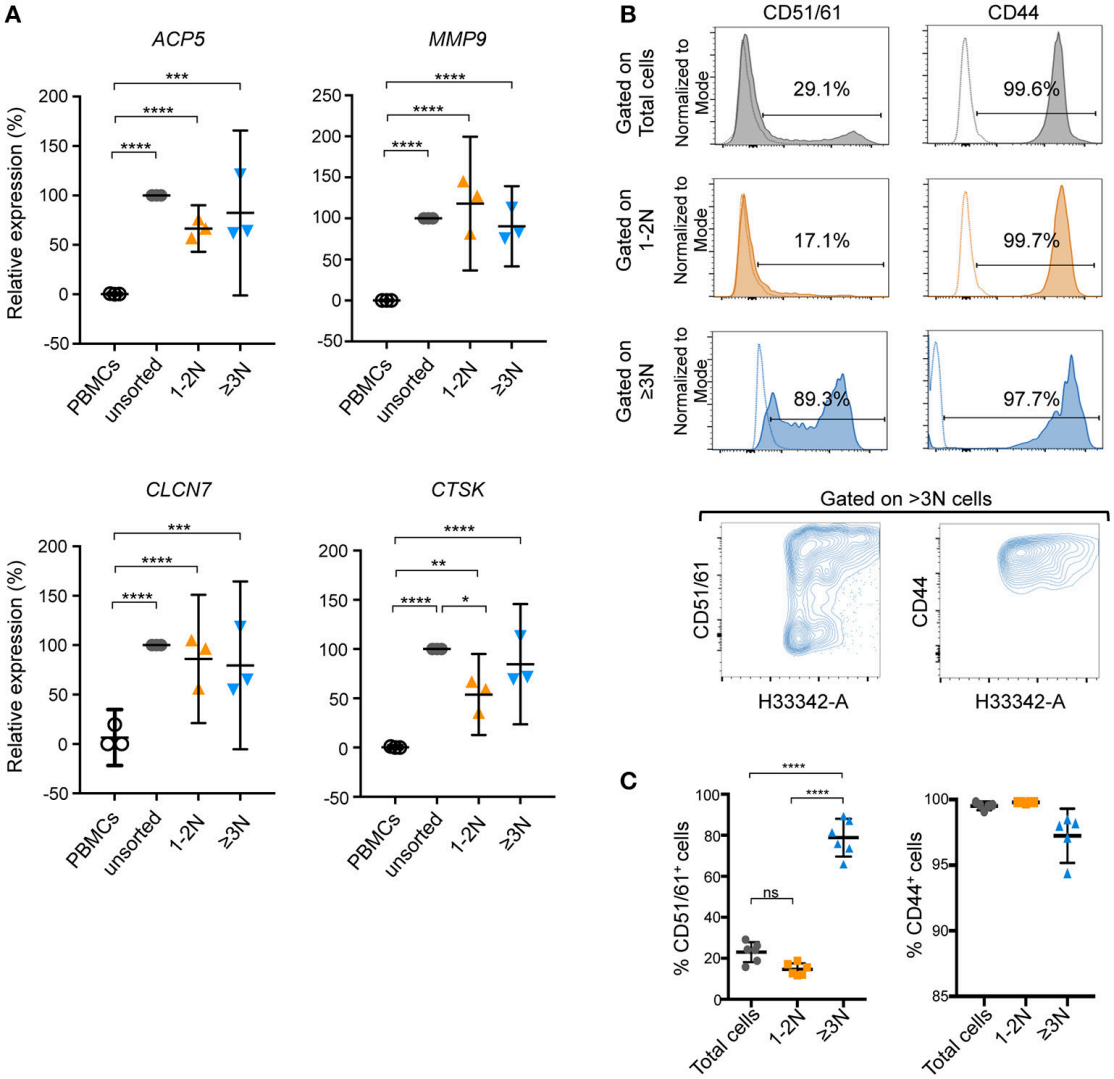
Focusing on pure human OCLs provides benefits for the analysis of OCL markers. **(A)** Gene expression of *ACP5, MMP9, CLCN7*, and *CTSK* was determined by RT-qPCR on unsorted and sorted (1-2N and ≥3N) human cells. Unsorted cells were defined as 100% of relative expression and contrasted to sorted cells. Results are represented as the mean with 95% confidence interval of three independent cell preparations and sortings, each from a pool of 2–3 donors, and experiments were conducted in triplicates. *ACP5*, tartrate-resistant acid phosphatase; *MMP9*, matrix metallopeptidase 9; *CLCN7*, chloride voltage-gated channel 7, *CTSK*, cathepsin K. **(B)** Flow cytometry analysis and **(C)** quantification of CD51/61^+^ and CD44^+^ human cells. Individual negative controls of each population are presented as dotted lines in the FACS histograms and positive events are given as a percentage. Total (gray) and 1-2N (orange) cells were compared to ≥3N (blue) cells. Data are representative as the means ±SEM of positive events (%) obtained from at least 5 independent cell preparations, each from 1 donor. ns, not significant; *p < 0.05; **p < 0.01; ***p < 0.001; ****p < 0.0001.

Nevertheless, human unsorted cells, sorted ≥3N cells and sorted 1-2N cells, as well as their precursors (PBMCs) could be analyzed by RT-qPCR for their expression of the OCL markers *ACP5, MMP9, CTSK*, and *CLCN7*. Compared to PBMCs, expression of all these markers was increased after OCL differentiation (Figure 6A). Except for *CTSK*, no significant differences in their expression level were evidenced when comparing unsorted and sorted populations (Figure 6A). This result suggested that human OCLs and the 1-2N cells growing with them are more related at the RNA expression level than murine ones.

Lastly, the Hoechst 33342 staining and gating strategy was applied on human OCLs for FACS analysis for the expression of two OCL markers involved in adhesion and podosome formation, CD51/61 (vitronectin receptor complex) and CD44 (23). As observed in murine cells, meaningful differences were observed in the expression of these two markers when gating on total or 1-2N cells compared to ≥3N OCLs (Figures 6B,C). In particular, the presence of OCLs with intermediate and high expression levels of these markers was noted, which are independent of the nuclei number (Figure 6B, lower panels). Again, no significant differences were observed between total cells or 1-2N cells because the large proportion of 1-2N cells overlaid the signals coming from multinucleated OCLs. Thus, as for murine OCLs, this observation indicates that the protein expression pattern of human OCLs is dramatically underestimated when analyzing a total population of differentiated cells compared to a pure population of OCLs.

## Conclusion

To date, the recurring problem in numerous studies focusing on OCLs is the difficulty to analyze pure populations of mature OCLs. As OCL-specific markers are equally expressed in low quantities by mononucleated precursor cells, they are unsuitable for the isolation of mature OCLs without a specific gating on multinucleated cells. However, since all cells have been stimulated with RANK-L and M-CSF, it is widely assumed that the mononucleated cells growing together with mature OCLs correspond to non-fused OCLs that already meet the criteria of mature OCLs. Consequently, many laboratories commonly analyze both populations together without any discrimination. Contrasting with this idea, we here demonstrated that murine 1-2N cells differ considerably from mature OCLs in their expression of OCL markers and their resorption capacity. Moreover, we showed that these 1-2N cells also differ from their CD11b^+^ precursors and, as their low expression of OCL markers indicates, only part of these cells is actually capable of further OCL differentiation. In human cells, although differences at the RNA level could not be demonstrated for the markers we analyzed, our results indicate that protein expression in 1-2N cells and unsorted cells differed significantly from OCLs, again demonstrating the great advantage of focusing on multinucleated cells. Therefore, excluding these non-OCL contaminating 1-2N cells provided a major interest as they represent up to 90% of analyzed cells, which lead to false-positive or inconclusive results concerning further OCL investigations.

In some reports, washing steps or enzymatic digestion have been implemented to discard the mononucleated cells that are supposed to be less attached to the plates than mature OCLs (7). Although OCL proportion is increased using such approaches, the degree of OCL purity is difficult to evaluate and may fluctuate considerably from one experiment to another. Besides the high reproducibility, flow cytometry represents a much more stringent strategy and allows for a much higher degree of OCL purity.

This approach has some limitations. Due to the difficulty to detach very large OCLs and the size of the sorter nozzle, the majority of the cells to be analyzed have <10 nuclei. The OCL number that can be recovered after sorting is about 1% of the cells seeded at the beginning of the experiment, meaning that, depending on the analysis to be performed, it may take large amounts of cells. And finally, although it greatly improves protein expression analysis in human OCLs, this procedure requires further optimization for functional analysis on human cells.

In conclusion, we here reported a novel, simple but robust technique for the isolation of pure OCLs by using a Hoechst dye for nuclear staining. Murine sorted OCLs can be analyzed for functional assays during several days after sorting, but not human OCLs in which the procedure dramatically reduces cell survival. However, using flow cytometry, reliable and reproducible investigations of pure OCL populations can be obtained, offering a tremendous advantage for OCL analysis and phenotyping. Additionally, using this innovative OCL isolation strategy unambiguously demonstrates for the first time the dramatic bias induced by analyzing murine unsorted cells instead of properly purified mature OCLs. This procedure also offers a huge benefit in the analysis of protein expression in human OCLs. Therefore, our findings may have important implications on the interpretation of numerous studies using unsorted OCLs for transcriptomic or proteomic analysis. The here presented isolation strategy will have high impact for clinicians and basic researchers working in the field of bone, osteoimmunology or focusing on the numerous bone and rheumatic diseases by unraveling several unanswered questions that could not be addressed up to now due to the lack of an efficient and reproducible OCL-sorting methodology. Thus, the here reported sorting and analysis approach represents a vitally important and efficient strategy to exclude impeding mononucleated cells and to consistently and reproducibly analyze pure and living murine osteoclasts using flow cytometry. Lastly, multinucleation is not a specificity of OCLs, but it can be observed in other monocytic cells. Therefore, this procedure is likely to be adapted for purification of other multinucleated cells such as those associated with malignant neoplasm, granuloma and infections (24).

## Author contributions

CB-W, AW, and M-BM: Study design; M-BM and LI: Performing experiment; M-BM, LI, AW, and CB-W: Data analysis; M-BM and CB-W: Drafting the manuscript; MR, AW, and CB-W: Revising the manuscript: M-BM, LI, MR, AW, and CB-W: Approving final version of manuscript.

### Conflict of interest statement

The authors declare that the research was conducted in the absence of any commercial or financial relationships that could be construed as a potential conflict of interest.
